# Effects of Possible Osteoporotic Conditions on the Recurrence of Chronic Subdural Hematoma

**DOI:** 10.3389/fneur.2020.538257

**Published:** 2020-09-24

**Authors:** Byeong Jin Ha, In-Suk Bae, Jae Min Kim, Jin Hwan Cheong, Je Il Ryu, Myung-Hoon Han

**Affiliations:** ^1^Department of Neurosurgery, Hanyang University Guri Hospital, Guri, South Korea; ^2^Department of Neurosurgery, Eulji University Hospital, Seoul, South Korea

**Keywords:** chronic subdural hematoma, Hounsfield unit, midline shift, diabetes, osteoporosis

## Abstract

The recurrence rate of chronic subdural hematoma (CSDH) has been reported to range from 2.3 to 33%. As bridging veins are composed of abundant collagen bundles and bone matrix, we aimed to investigate the possible associations between skull Hounsfield unit (HU) values and the recurrence of CSDH. We retrospectively enrolled patients with CSDH who underwent burr hole surgery. The HU values of the frontal skull were measured on brain CT scans. The cumulative hazard for recurrence was estimated according to predictive factors. To identify the independent predictors associated with the recurrence of CSDH, hazard ratios (HRs) were estimated using multivariate Cox regression analysis. A total of 208 consecutive patients who underwent burr hole trephination for CSDH over a 7-years period at a single institution were enrolled in this study. We found that age, greater midline shift (≥10.5 mm), lower skull HU (<769.5), and diabetes were independent predictors for the recurrence of CSDH (HR 1.06, 95% confidence interval [CI] 1.00–1.12, *p* = 0.042; HR 5.37, 95% CI 1.48–19.46, *p* = 0.010; HR 6.71, 95% CI 1.84–24.45, *p* = 0.004; and HR 3.30, 95% CI 1.05–10.43, *p* = 0.042, respectively). A relationship between possible low bone mineral density (BMD) and CSDH recurrence was observed. In addition, age, greater preoperative midline shift, and diabetes were also identified as predictive factors for recurrence. We expect that our findings may facilitate our understanding of the possible association between CSDH and BMD.

## Introduction

Chronic subdural hematoma (CSDH) is a common condition in elderly people. The recurrence rate of CSDH has been reported to range from 2.3 to 33% (1). Previous studies have reported many risk factors for CSDH recurrence, including brain atrophy, underexpansion, coagulopathy or anticoagulant use, male sex, hypertension, diabetes, bilateral CSDH, large preoperative hematoma volume, septations, mixed density of hematoma on brain computed tomography (CT) scans, iso- or hypointensity on T1-weighted magnetic resonance images, preoperative midline shift, persistence of a mass effect after burr hole trephination, large postoperative residual hematoma, and postoperative pneumocephalus (2, 3).

To our knowledge, no study has investigated the association between skull Hounsfield unit (HU) values calculated from CT scans and recurrence in patients who have undergone burr hole trephination for CSDH. It is well-accepted that CSDH is caused by the tearing of bridging veins with subsequent bleeding (4). We initiated this study based on the premise that bridging veins are composed of abundant collagen bundles and smooth muscle cells, which are composed of type 1 collagen as well as bone matrix (5, 6). Therefore, we hypothesized that a low bone mineral density (BMD) would negatively influence the integrity of bridging veins and bone. We conjectured that this mechanism may be associated with the recurrence of CSDH after the initial burr hole surgery. We previously reported the significant correlation between skull HU values and BMD (7). Therefore, to assess this hypothesis, frontal skull HU values were measured on brain CT scans of all study patients. Further, we examined the relationship between skull HU values and CSDH recurrence.

## Materials and Methods

### Study Patients

Using the database of the Traumatic Brain Injury Registry of our hospital, we retrospectively investigated all consecutive patients who were diagnosed with CSDH and underwent burr hole surgery from January 1, 2012, to December 31, 2018. We excluded patients (i) with iatrogenic error or surgical complication, (ii) with no initial brain CT scan or with missing data, and (iii) with no measurable intercortical spongy bone in the frontal skull on brain CT. Recurrence was defined as the (i) presence of newly developed CSDH in the ipsilateral subdural space of the initial burr hole surgery on follow-up brain CT scan, (ii) development of neurologic deficits, and (iii) requirement for reoperation.

This study was approved by the Institutional Review Board of Hanyang University Guri Hospital, Korea, and performed in accordance with the tenets of the Declaration of Helsinki. Due to the retrospective nature of the study, the requirement for informed consent was waived. All individual records were anonymized before the analysis.

### Surgical Procedures and Management

One- or two-burr hole craniostomy was performed in a standardized manner with or without saline irrigation under general anesthesia. All patients with bilateral CSDH underwent burr hole craniostomy of both sides simultaneously in a single operation. A closed-system drainage was inserted in all patients, and the drain was placed ~30–50 cm below the patient's head level. The drain was removed within 3 days in most cases. According to our policy and protocol, preoperative dexamethasone was not used in any case. Reoperation was performed with the same procedure in patients with recurrence. Antiplatelet or anticoagulant agents were discontinued before surgery and usually restarted after 1 month from the day of surgery. We usually performed follow-up CT examinations immediately and 1 week after surgery. Patients showing no complications were generally discharged within 10 days.

### Clinical and Radiographic Variables

We investigated the factors possibly associated with recurrence in patients with CSDH after burr hole surgery. The demographics, clinical information, and operative information of the enrolled patients were investigated by two trained research members using electronic medical records. Clinical data including sex, age, side of operation, reoperation, hypertension, diabetes, chronic kidney disease, alcohol intake, and history of antithrombotic agent use were collected from medical and operative records.

We analyzed the initial CT scans of all study patients. All radiologic findings were confirmed by two faculty neurosurgeons (I-SB and BH) blinded to the clinical data using the picture archiving and communication system (PACS). Radiographic variables, including the types of the internal architecture of the hematoma, midline shift, and hematoma volume, were evaluated using the initial preoperative CT scans. The internal architecture of the hematoma was classified into four types: homogeneous, laminar, separate, and trabecular (1). The laminar, separate, and trabecular types were also categorized into a heterogeneous group for the analysis. We calculated the hematoma volume using the ABC/2 technique (A = length [in centimeters] between each corner of the subdural crescent, B = width as the maximum thickness [in centimeters] of the hematoma from the inner table of the skull perpendicular to the length, C = depth multiplying the number of slices on which the hematoma was visible by the slice thickness listed on the CT scan) (8). The method and reliability of the ABC/2 technique for subdural hematoma measurement on brain CT scans have been described elsewhere (9).

### Measurement of Frontal Skull HU

**Figure 1:**
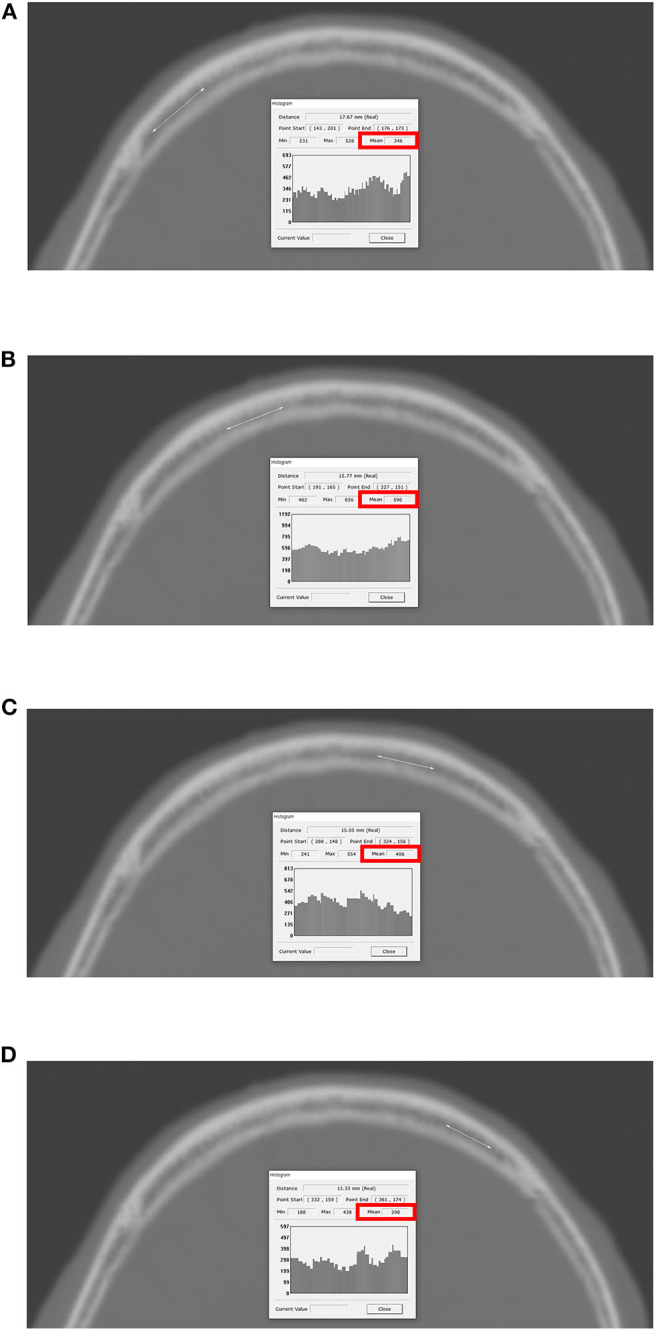
Measurement of HU values at each of four lines on the frontal bone. The PACS automatically calculates the maximum, minimum, and mean HU values according to the values on the drawn lines, and the mean HU value on each of the four lines was recorded. **(A)** Right lateral; **(B)** right medial; **(C)** left medial; **(D)** left lateral. HU, Hounsfield unit; PACS, picture archiving and communication system.

All CT scans (4.0- to 5.0-mm slice thicknesses) were obtained with CT scanners (Siemens Flash 64, München, Germany) at our hospital. Birnbaum BA et al. described that the variations in HU values are very small (range of 0–20 HU) between five CT scanners, including Siemens (10). The detailed methods for measuring HU values at each of four lines on the frontal skull were previously described precisely (Figure 1) (7). The maximum, minimum, and mean HU values were automatically calculated by the PACS based on the values corresponding to the drawn lines, and the mean HU values were recorded for the study. To reduce measurement errors, all brain CT scans were magnified for HU measurement. The frontal skull HU was measured by a faculty neurosurgeon (I-SB) blinded to the clinical data in all study patients.

### Statistical Methods

We expressed continuous variables as means ± standard deviations or medians with interquartile ranges. Discrete variables are expressed as counts and percentages. The chi-square test and Student's *t*-test were used to identify differences between the non-recurrence and recurrence groups. The mean frontal skull HU values used in all analyses were calculated as follows: (mean right lateral HU + mean right medial HU + mean left medial HU + mean left lateral HU)/4.

Box plots were used to show the association among the midline shift, mean frontal skull HU values, and recurrence. Receiver operating characteristic curve analysis was performed to identify whether the midline shift and frontal skull HU can predict recurrence after burr hole surgery for CSDH.

The cumulative hazard for recurrence was evaluated using the Kaplan–Meier method classified based on several predictive factors, with censoring of patients who exhibited no recurrence or recurrence-related symptoms on the last follow-up CT scan or visit during the follow-up period. According to previous studies, we set the end point of the current study at 1 year (365 days) from the first burr hole surgery for CSDH (11, 12). The time interval to recurrence was defined as the number of days between the first burr hole trephination and the recurrence of CSDH requiring reoperation based on brain CT scans. We then calculated hazard ratios (HRs) with 95% confidence intervals (CIs) using Cox regression analyses. These values were used to identify independent predictive factors associated with recurrence after burr hole surgery in patients with CSDH. Values of *p* < 0.05 were considered statistically significant.

All statistical analyses were performed using R version 3.5.2 (https://www.r-project.org/).

## Results

### Characteristics of the Study Patients

A total of 208 consecutive patients (>18 years old) who underwent burr hole trephination for CSDH over a 7-years period at our hospital were finally enrolled in the study. In total, 19 patients (9.1%) were reoperated for recurrence of CSDH within 1 year from the initial burr hole surgery for CSDH. The mean patient age was 69.9 years, and 63.9% of the patients were men. A total of 26 patients (12.5%) underwent bilateral burr hole trephinations. The mean preoperative midline shift was 10.2 mm, and the mean hematoma volume was 120.5 cm^3^. Further descriptive data are shown in Table 1.

**Table 1:** Characteristics of patients with chronic subdural hematoma at our hospital.

**Characteristics**	**Non-recurrence**	**Recurrence**	**Total**	***p***
Number (%)	189 (90.9)	19 (9.1)	208 (100)	
Sex, male, *n* (%)	121 (64.0)	12 (63.2)	133 (63.9)	0.940
Age, mean ±*SD*, years	69.6 ± 12.4	73.2 ± 9.4	69.9 ± 12.2	0.221
Age group, *n* (%)				
≥ 65 years	125 (66.1)	16 (84.2)	141 (67.8)	0.108
Side of operation, *n* (%)				0.585
Right	69 (36.5)	7 (36.8)	76 (36.5)	
Left	95 (50.3)	11 (57.9)	106 (51.0)	
Bilateral	25 (13.2)	1 (5.3)	26 (12.5)	
Internal architecture of the hematoma, *n* (%)				0.221
Homogeneous	66 (34.9)	6 (31.6)	72 (34.6)	
Laminar	26 (13.8)	0 (0)	26 (12.5)	
Separate	56 (29.6)	6 (31.6)	62 (29.8)	
Trabecular	41 (21.7)	7 (36.8)	48 (23.1)	
Preoperative midline shift, mean ±*SD*, mm	10.0 ± 3.8	12.0 ± 3.0	10.2 ± 3.8	0.024
Preoperative midline shift, median (IQR), mm	10.2	11.5	10.5	0.024
	(7.1–12.8)	(10.8–13.9)	(7.4–12.8)	
Preoperative hematoma volume, mean ±*SD*, cm^3^	120.4 ± 47.4	121.3 ± 42.9	120.5 ± 47.0	0.933
Preoperative hematoma volume, median (IQR), cm^3^	113.4	118.7	113.7	0.933
	(87.6–144.8)	(92.2–139.9)	(88.0–144.2)	
Past medical history, *n* (%)				
Hypertension	100 (52.9)	11 (57.9)	111 (53.4)	0.678
Diabetes	43 (22.8)	8 (42.1)	51 (24.5)	0.062
Chronic kidney disease	6 (3.2)	1 (5.3)	7 (3.4)	0.630
Alcohol	73 (38.6)	7 (36.8)	80 (38.5)	0.879
Antithrombotic				0.145
Antiplatelet	52 (27.5)	10 (52.6)	62 (29.8)	
Anticoagulant	3 (1.6)	0 (0)	3 (1.4)	
Both	1 (0.5)	0 (0)	1 (0.5)	

*SD, standard deviation; IQR, interquartile range*.

### Skull HU Values According to CSDH Recurrence

Table 2 shows the significant differences in the mean frontal skull HU values according to CSDH recurrence. The overall average mean frontal skull HU value was 763.7 in all study patients and was 772.3 in the non-recurrence group and 677.4 in the recurrence group.

**Table 2:** Descriptive statistics of mean frontal skull HU values according to recurrence in patients with chronic subdural hematoma.

**Characteristics**	**Non-recurrence**	**Recurrence**	**Total**	***p***
Overall mean frontal skull HU value, median (IQR)	780.5	723.8	769.5	0.009
	(667.8–891.3)	(574.8–768.5)	(660.9–888.4)	
Overall mean frontal skull HU value, mean ±*SD*	772.3 ± 149.0	677.4 ± 155.4	763.7 ± 151.7	0.009
**Mean HU value at each of four sites in the frontal skull, mean** **±*****SD***				
Right lateral	733.7 ± 148.9	642.6 ± 135.8	725.4 ± 149.8	0.011
Right medial	807.0 ± 172.0	710.7 ± 175.5	798.2 ± 174.1	0.021
Left medial	806.4 ± 147.5	691.3 ± 160.3	795.9 ± 152.0	0.002
Left lateral	742.2 ± 161.1	665.1 ± 164.4	735.2 ± 162.5	0.048
Average, medial	806.7 ± 156.0	701.0 ± 165.4	797.0 ± 159.4	0.006
Average, lateral	738.0 ± 148.9	653.8 ± 149.3	730.3 ± 150.5	0.020

*HU, Hounsfield unit; IQR, interquartile range; SD, standard deviation*.

### Association Between Preoperative Midline Shift and Skull HU and CSDH Recurrence

**Figure 2:**
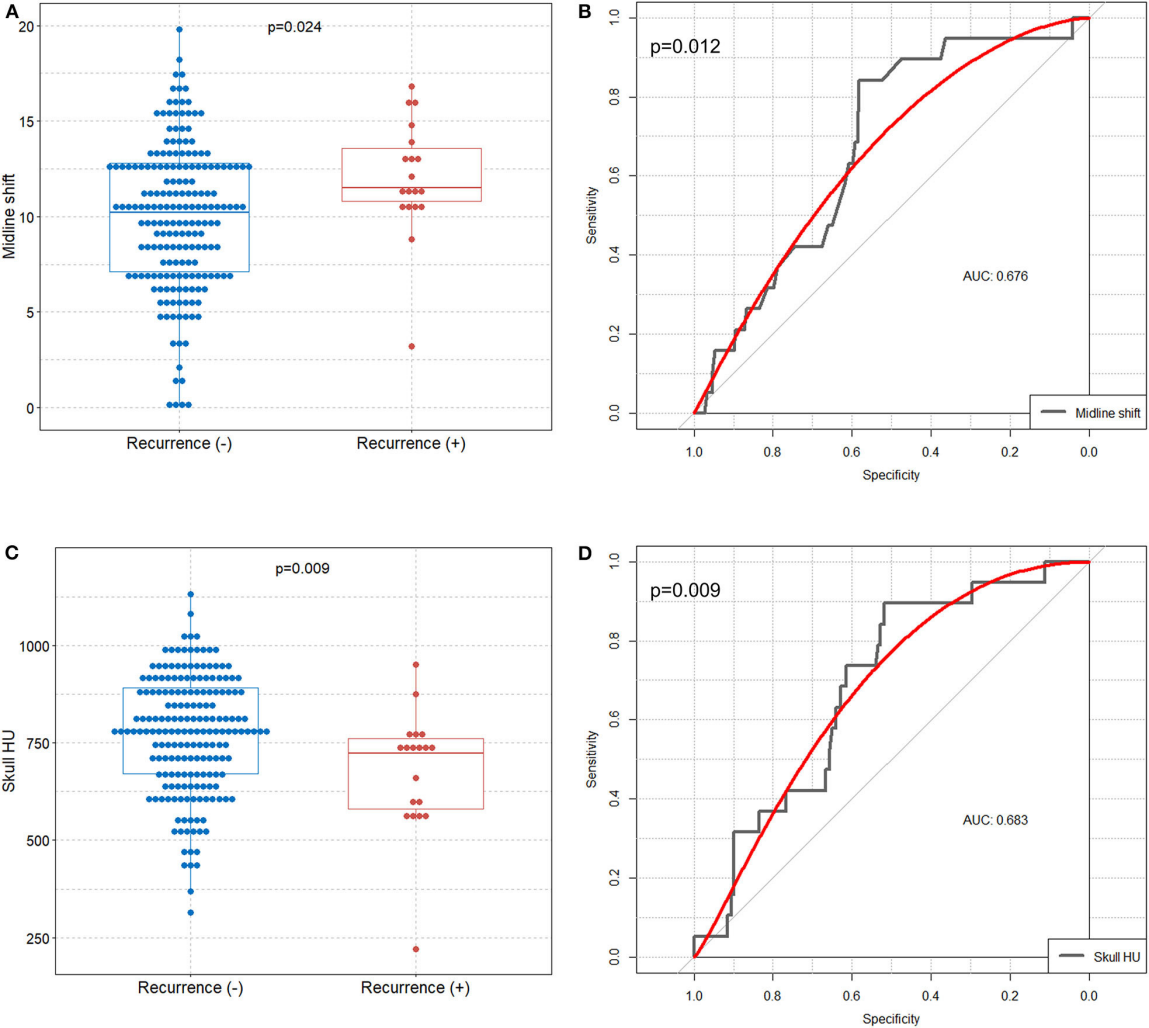
Comparison of preoperative midline shift and mean frontal skull HU values between the recurrence and non-recurrence groups, and identification of the preoperative midline shift and skull HU values for predicting CSDH recurrence. Boxplots with dot plots of **(A)** preoperative midline shift and **(C)** mean frontal skull HU values according to recurrence. Receiver operating characteristic curves to identify the **(B)** preoperative midline shift and **(D)** mean frontal skull HU values for predicting recurrence. HU, Hounsfield unit; CSDH, chronic subdural hematoma; AUC, area under the curve.

Boxplot analysis revealed a significantly higher preoperative midline shift and lower mean frontal skull HU values in the recurrence group than in the non-recurrence group (Figures 2A,C). In receiver operating characteristic curve analysis, we identified the preoperative midline shift (area under the curve = 0.676, *p* = 0.012) and mean frontal skull HU (area under the curve = 0.683, *p* = 0.009) as predictive values for CSDH recurrence (Figures 2B,D).

### Cumulative Hazard of CSDH Recurrence According to Predictive Factors

**Figure 3:**
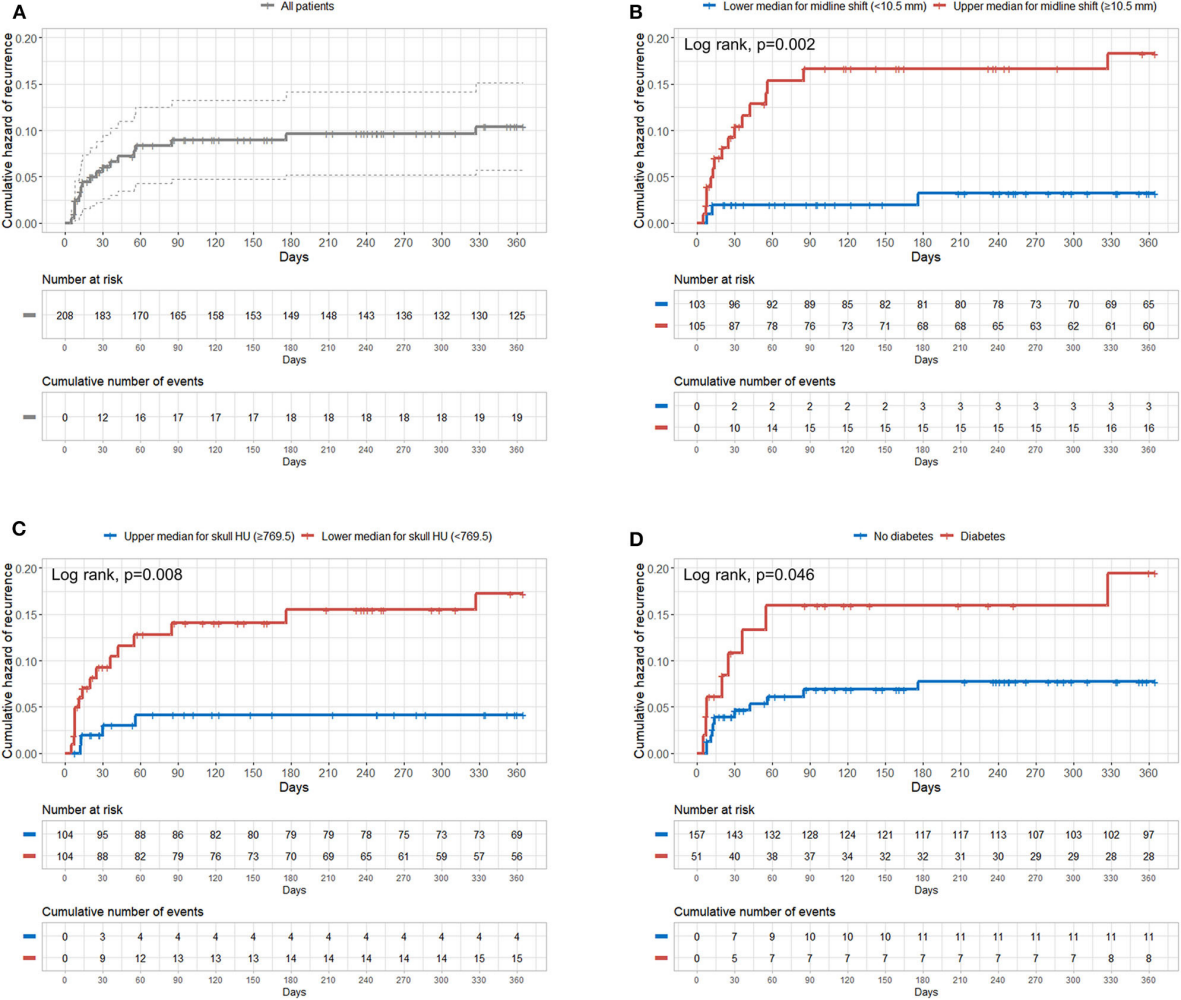
Cumulative hazard of recurrence according to preoperative midline shift, mean frontal skull HU values, and diabetes. **(A)** Overall; **(B)** median of the preoperative midline shift (10.5 mm); **(C)** median of mean frontal skull HU values (769.5); **(D)** diabetes. HU, Hounsfield unit.

Figure 3A shows the overall cumulative hazard of CSDH recurrence within 1 year from the initial burr hole trephination for CSDH. We found that patients in the upper median for preoperative midline shift (≥10.5 mm), those in the lower median for skull HU (<769.5), and those with diabetes showed significantly higher recurrence rates of CSDH (Figures 3B–D).

### Independent Predictive Factors for CSDH Recurrence

Multivariate Cox regression analysis identified age, greater midline shift (≥10.5 mm), lower skull HU (<769.5), and diabetes as independent predictors for CSDH recurrence (HR 1.06, 95% CI 1.00–1.12, *p* = 0.042; HR 5.37, 95% CI 1.48–19.46, *p* = 0.010; HR 6.71, 95% CI 1.84–24.45, *p* = 0.004; and HR 3.30, 95% CI 1.05–10.43, *p* = 0.042, respectively) (Table 3). Although the study patients showed a relatively narrow age range (most patients were elderly, mean age = 69.9 years), age was negatively associated with BMD. Therefore, we performed multivariate Cox regression with adjustment for age as a continuous variable. In addition, we observed no significant correlation between age and skull HU values in the study patients (*B* = 0.678, *p* = 0.434) (Supplementary Figure).

**Table 3:** Univariate and multivariate Cox regression analyses of the association between recurrence and various variables in patients with chronic subdural hematoma.

**Variable**	**Univariate analysis**		**Multivariate analysis**	
	**HR (95% CI)**	***p***	**HR (95% CI)**	***p***
**Sex**				
Male	Reference		Reference	
Female	1.07 (0.42–2.71)	0.890	2.06 (0.70–6.03)	0.188
Age (per 1-year increase)	1.03 (0.99–1.07)	0.167	1.06 (1.00–1.12)	0.042
**Internal Architecture of the Hematoma**				
Homogeneous	Reference		Reference	
Heterogeneous	1.23 (0.47–3.24)	0.675	0.62 (0.22–1.75)	0.364
**Midline Shift**				
Lower median (<10.5 mm)	Reference		Reference	
Upper median (≥10.5 mm)	5.74 (1.68–19.79)	0.005	5.37 (1.48–19.46)	0.010
**Mean Frontal Skull HU**				
Lower median (<769.5)	4.00 (1.33–12.07)	0.014	6.71 (1.84–24.45)	0.004
Upper median (≥769.5)	Reference		Reference	
Initial hematoma volume (per 1 cm^3^ increase)	1.00 (0.99–1.01)	0.943	1.01 (0.99–1.02)	0.404
**Side of Operation**				
Unilateral	2.79 (0.37–20.88)	0.319	7.71 (0.54–109.84)	0.132
Bilateral	Reference		Reference	
**Past Medical History**				
Hypertension	1.20 (0.48–2.98)	0.697	0.44 (0.13–1.45)	0.175
Diabetes	2.45 (0.99–6.10)	0.054	3.30 (1.05–10.43)	0.042
Chronic kidney disease	1.88 (0.25–14.11)	0.538	4.41 (0.40–48.42)	0.225
Alcohol	0.87 (0.34–2.20)	0.760	2.44 (0.73–8.14)	0.147
Antithrombotic	2.40 (0.98–5.90)	0.057	2.39 (0.78–7.31)	0.127

*HR, hazard ratio; CI, confidence interval; HU, Hounsfield unit*.

## Discussion

We found that the group with a possible lower BMD had an ~6.7-fold higher risk of CSDH recurrence than the possible higher BMD group after adjusting for other predictive factors including age. In addition, older age, greater preoperative midline shift, and diabetes were independent predictors for CSDH recurrence. To our knowledge, this study is the first to suggest a possible relationship between possible lower BMD and recurrence of CSDH.

Previous studies have demonstrated that specific regional cancellous bone HU values from CT scans show a strong correlation with the T-score and may be useful for predicting osteoporotic conditions (13–15). We previously showed a strong correlation between the T-score and frontal skull HU values (7, 16). Because osteoporosis is a systemic disease and is strongly related to genetic components of type 1 collagen (*COL1A1* and *COL1A2)*, we propose that osteoporotic conditions may influence cancellous bone of the skull (17).

It is well-known that type 1 collagen is a major bone component. A previous study reported that the bridging vein wall is composed of collagen bundles, with a volume fraction of ~61% (6). In addition, smooth muscle cells are also associated with cortical bridging veins (5, 18). The smooth muscle cell is also composed of collagen type 1 (19). Therefore, on the basis of the above findings and assumptions, we postulated that an osteoporotic condition, which shows a strong genetic association, leading to systemic disease may also negatively influence the integrity of the bridging veins as well as bone because both bone and bridging veins are associated with type 1 collagen tissues. Weaker bridging veins may be more vulnerable to rupture, and this may naturally lead to a higher chance of recurrence of CSDH. Therefore, we believe that recurrence rate of CSDH may be higher in patients with osteoporotic conditions. In addition, we recently showed an association between osteoporosis and cerebral atrophy (20). Preexisting cerebral atrophy is associated with alteration of brain elasticity and causes cerebral under-expansion after burr hole surgery for CSDH (21, 22). Moreover, this may lead to a persistent cavity in the subdural space and subsequently cause CSDH recurrence (2).

In the present study, patient age, greater preoperative midline shift, and diabetes were also predictors of recurrence. Cerebral atrophy occurs during the normal aging process. Therefore, older patients tend to experience a recurrence of CSDH because of the persistent space between the subdural and arachnoid layers resulting from cerebral atrophy (23). The preoperative and postoperative midline shift values have already been considered as predictive factors for CSDH recurrence (3, 22, 24, 25). The sudden decrease in intracranial pressure after trephination may lead to a rapid expansion of the brain parenchyma, which consequently induces stress on the surrounding vessels and a higher risk of acute rebleeding (24). Several previous studies have reported that diabetes is associated with the recurrence of CSDH (22, 26). Exudation due to capillary vasculopathy caused by diabetes or well-developed neovascularization of the neomembrane in diabetes patients may play an important role in the recurrence of CSDH (27). It has also been reported that type 2 diabetes is associated with an increased risk of brain atrophy (28, 29). The cerebral atrophy induced by type 2 diabetes is naturally thought to be associated with the recurrence of CSDH as described above. The association between antithrombotic use and the recurrence of CSDH was slightly short of being significant in this study; however, a previous meta-analysis reported that antithrombotic drugs increased the risk of CSDH recurrence (30). Use of antithrombotic drugs may raise the possibility of micro-bleeding, and this may accelerate the growth of hematoma and recurrence of CSDH.

This study had several limitations. First, our study had a retrospective and single-center design. Our findings may be less accurate than those from a planned prospective study, which limits the generalizability of our results. Second, HU measurement errors may have occurred. However, we magnified all brain CT scans for HU measurement and initially excluded patients with no measurable cancellous bone of the frontal skull on brain CT, as described in the Methods. Third, skull HU is not the actual T-score. The actual T-score was not available because patients with CSDH usually do not undergo BMD testing. However, we have previously shown that the skull HU values have relatively high specificity and sensitivity in predicting the actual T-score (7).

In conclusion, our study suggests the existence of a relationship between possible low BMD and recurrence of CSDH. In addition, our results revealed that age, greater preoperative midline shift, and diabetes are also predictors for recurrence. Our findings may be useful for predicting recurrence in the clinical course of CSDH. Further, we expect that our findings may help enhance the understanding of the underlying mechanism of the association between CSDH and BMD in the future.

## Data Availability Statement

The raw data supporting the conclusions of this article will be made available by the authors, without undue reservation, to any qualified researcher.

## Ethics Statement

The studies involving human participants were reviewed and approved by the Institutional Review Board of Hanyang University Guri Hospital, Korea. The ethics committee waived the requirement of written informed consent for participation.

## Author Contributions

M-HH: conception, design of the study, analysis of data, and visualization. BH and I-SB: acquisition of data. BH and M-HH: manuscript writing. JK, JC, and JR: study supervision, reexamination, and revision of the paper. All authors read and approved the final manuscript.

## Conflict of Interest

The authors declare that the research was conducted in the absence of any commercial or financial relationships that could be construed as a potential conflict of interest.

## Abbreviations

CSDH: chronic subdural hematoma
HU: Hounsfield unit
BMD: bone mineral density
PACS: picture archiving and communication system
HR: hazard ratio
CI: confidence interval
AUC: area under the curve.
