# Supplementary material for: Effects of Possible Osteoporotic Conditions on the Recurrence of Chronic Subdural Hematoma
Source: Front Neurol. 2020 Sep 24;11:538257. doi: 10.3389/fneur.2020.538257 (PMC7542308; doi:10.3389/fneur.2020.538257)
Supplement: Supplementary file 1 [file Table_1.DOCX]

Supplementary Material

# Supplementary Figure


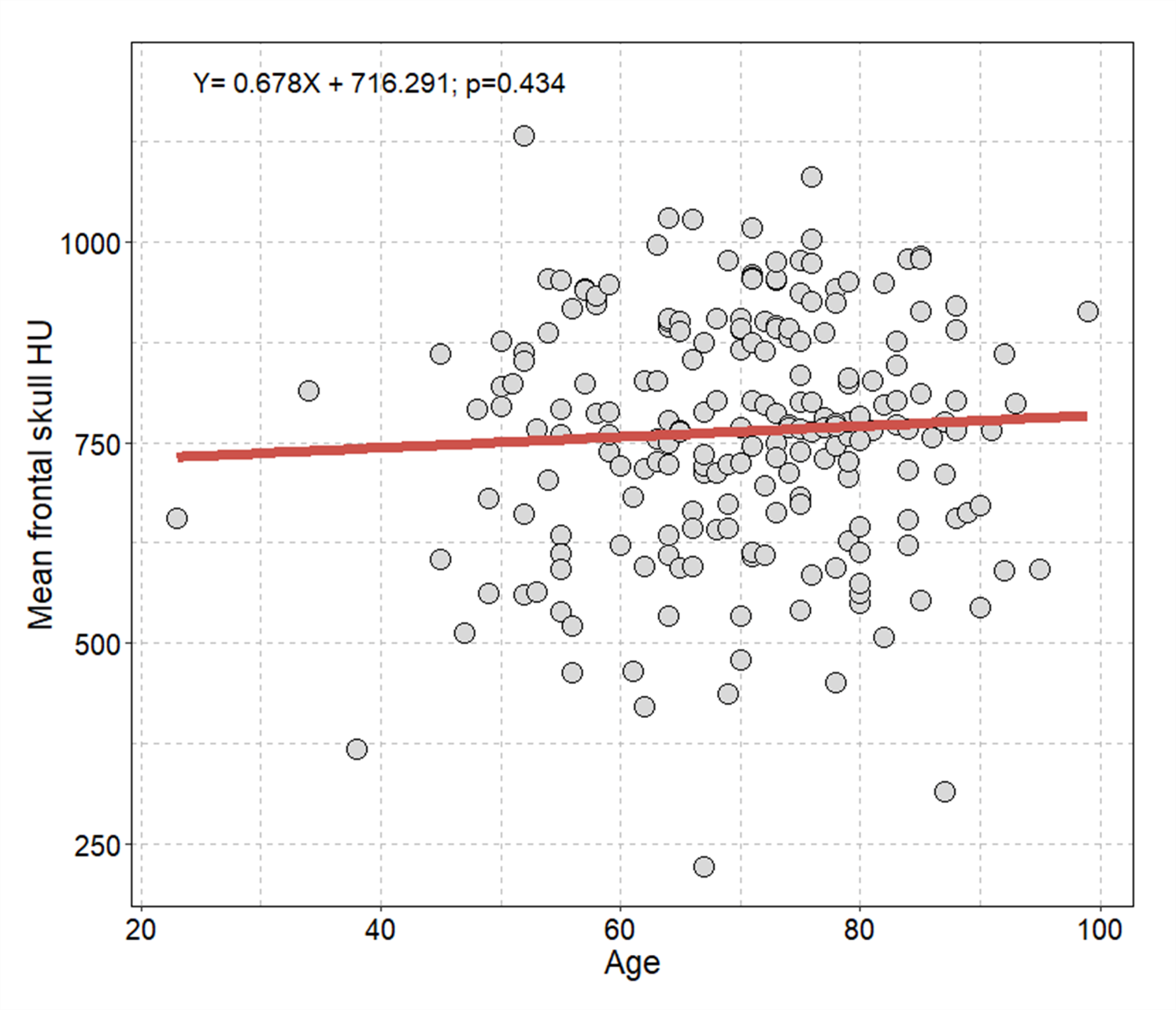


**Supplementary Figure.** Scatterplot with linear regression line showing the association between age and mean frontal skull HU values. HU=Hounsfield unit..
